# Lymph Node Ratio (LNR) Discriminates Prognostication in pN1a-b and pN2 Stage-III Colon Cancer

**DOI:** 10.7150/jca.104336

**Published:** 2025-01-01

**Authors:** Erman Akkus, Mehmet Kayaalp, Beliz Bahar Karaoğlan, Cihangir Akyol, Güngör Utkan

**Affiliations:** 1 Ankara University Faculty of Medicine, Department of Medical Oncology, Ankara, Türkiye.; 2 Ankara University Cancer Research Institute, Ankara, Türkiye.; 3 Ankara University Faculty of Medicine, Department of General Surgery, Ankara, Türkiye.

**Keywords:** colon cancer, lymph node, lymph node ratio, stage, prognosis

## Abstract

**Background:** The lymph node ratio (LNR), involved nodes/ lymph nodes examined, is associated with survival in colon cancer. Previous studies investigated the prognostic role of LNR regardless of TNM N staging or compared LNR and TNM N stages for prognostic strength. However, LNR may be utilized to obtain additional prognostic information rather than replacing TNM staging in daily practice. This study aimed to evaluate the role of LNR in TNM N stages to provide further prognostic information in daily practice.

**Methods:** Patients with stage-III colon cancer who underwent surgery and adjuvant chemotherapy were included. pN1c tumors (tumor deposits without node involvement) and rectal cancers were excluded. Clinicopathological parameters and LNR in pN1a-b and pN2 groups were evaluated for recurrence-free survival (RFS).

**Results:** A total of 97 patients were included [pN1a-b: n=69 (71.1%) and pN2: n=28 (28.9%)]. Median LNR in the entire population was 0.09 (0.01-0.84) with a median lymph node examined of 22 (8-89) and involved of 2 (1-17). Median RFS was not reached in the pN1a-b and pN2 groups during a median follow-up of 20.8 months (1.13-101.03), with significantly better survival of the pN1a-b group (p=0.003). Among the pN1a-b group, the LNR cut-off was set as 0.10. LNR significantly discriminated RFS (Median not-reached, p=0.001). Among the pN2 group, the LNR cut-off was set as 0.25 and LNR significantly discriminated RFS [Not reached vs. 11.40 months (95%CI: 3.57-16.83), p=0.004]. Combined pN-LNR groups revealed significant discrimination in RFS (p<0.001). RFS was not statistically different between pN2-LNR≤0.25 and pN1-LNR>0.10 groups (p=0.282). In multivariable analysis with clinicopathological parameters, only LNR was significant (p=0.023), whereas the pN stage did not remain significant (p=0.637).

**Conclusion:** LNR adds further prognostication in pN1a-b and N2 groups. LNR may be utilized to detect patient subgroups in different TNM N sages (pN1a-b and pN2) but with similar prognoses. This further prognostic information may assist clinical decisions in practice. The results of this study emphasize an adequate and higher number of lymph node samples in surgery.

## Introduction

Colorectal cancer (CRC) represents the third most common cancer and the second most common cause of cancer mortality worldwide^1^. Surgical treatment is the mainstay for the management of localized disease. In the 8th TNM system used in colorectal cancer staging, lymph node positivity increases the disease to stage III and above, and there are significant differences in prognosis and life expectancy between lymph node-negative and positive patients. Reported 5-year survival rates following surgical resection alone are 99% for stage I, between 68% and 83% for stage II, and between 45% and 65% for stage III. Thereby, lymph node positivity is one of the main markers affecting the adjuvant chemotherapy decision^2^.

Lymph node positivity is classified as N1 and N2 in the 8th TNM classification. N1a is one regional lymph node positivity, N1b is two or three regional lymph nodes positivity, N2a is four to six regional lymph nodes positivity, and N2b seven or more regional lymph nodes are positivity. N1c is defined as positive tumor deposits in subserosa, mesenteric, nonperitonealized pericolic, or perirectal/mesorectal tissues without positive lymph nodes^2^. Performing an adequate and appropriate surgery determines the success of treatment in localized colorectal cancer and it is recommended to dissect at least 12 lymph nodes, affecting adjuvant treatment decisions and prognosis in stage II disease^3^. In stage III, on the other hand, the lymph node ratio (LNR) has been investigated as a prognostic factor, which is calculated by dividing the number of metastatic lymph nodes by the total number of lymph nodes harvested and examined^4^.

Several studies have reported the prognostic value of LNR; however, those studies are designed to analyze the prognosis of LNR categories without combining the TNM N stage^5^-^12^. Nevertheless, it may not be convenient to consider solely LNR for prognostic assessment without taking TNM N staging into account in daily practice. Thereby, this study aims to evaluate the role of LNR in TNM N stages to provide further prognostication for assisting daily practice.

## Methods

### Patients and data

The retrospective cohort data of Ankara University Faculty of Medicine Localized Colorectal Cancer Cohort (AUTF-NMKRK) including 432 patients with newly diagnosed localized CRC between 2015-2024, was utilized. Inclusion criteria were listed for patients: > 18 years, both genders, newly diagnosed, histopathologically confirmed, non-metastatic lymph node-positive colon adenocarcinoma. Exclusion criteria were listed: Lymph node-negative patients, pN1c tumors (only tumor deposits without regional lymph node involvement), M1, and rectal cancers.

Age, gender, comorbidities [diabetes, hypertension, coronary artery disease (CAD)], primary tumor location, presence of urgent surgery, T and N stages (according to AJCC 8th edition,2017), histopathological features [grade, mucinous component, lymphovascular invasion (LVI), perineural invasion (PNI), tumor deposits, budding, total examined lymph node number], microsatellite instability status (MSI), *RAS-RAF* mutational status, ABO and Rh blood groups, adjuvant treatments, recurrence and survival data were recorded. All data were retrieved from the Avicenna Hospital Data Management System.

Recurrence-free survival (RFS) was defined as the time between surgery and the first disease recurrence or death.

Ethical approval was obtained from the Clinical Research Ethics Committee of Ankara University Faculty of Medicine (Number: 2022000659, 2022/659 and Number:2023000367-1, 2023/367) in compliance with the Declaration of Helsinki.

### Outcomes

Characteristics of the entire study population, pN1 and pN2 groups were presented and compared between N1 and N2 groups. RFS of pN1 and pN2 groups were presented.

The lymph node ratio (LNR) was defined as the number of involved nodes/numbers of lymph nodes examined histopathologically. Repeated log-rank tests providing best discrimination determined LNR cut-offs in pN1 and pN2 groups. RFS according to the LNR cut-offs in pN1 and pN2 groups were calculated. Clinicopathological parameters and LNR were multivariably analyzed for RFS.

Primary endpoints were the prognostic value of LNR in the pN1 and pN2 groups. The secondary endpoint was prognostic similarity in LNR subgroups of pN1 and pN2 stages.

### Statistical analysis

Continuous variables were given as median [minimum (min)-maximum (max)], and categorical variables were presented as the percentage. Univariable comparisons were performed using chi-square, Fisher exact, Student's t, Mann-Whitney U tests, and Cox regression, where needed. The statistically significant variables in the univariable analysis were included in the multivariable analysis. Cox regression analysis was utilized for multivariable analyses. Survivals were estimated by the Kaplan-Meier method and compared by log-rank test. All p-values were based on a 2-tailed test of significance (p=0.05). Statistical analyses were conducted using the MedCalc® Statistical Software version 22.026 (MedCalc Software Ltd, Ostend, Belgium).

## Results

### Clinicopathological and survival characteristics of patients

A total of 97 patients were included. 71.1% of patients (n=69) had pN1a-b and 28.9% (n=28) N2 disease. Median age in the entire population was 67 (21-94), and 58.8% (n=57) were male. T stage was mainly pT3 (n=70, 72.2%), and 54.6% (n=53) of the tumors were left-sided. Mucinous component (14.5% vs. 35.7%, p=0.019), lymphovascular invasion (43.5% vs 67.9%, LVI) (p=0.030), perineural invasion (PNI) (26.1% vs. 53.6%, p=0.010), budding (15.9% vs. 42.9%, p=0.005), and MSI-H tumors (1.4% vs. 14.3%, p=0.025) were more common in the pN2 group compared to the pN1a-b. Adjuvant chemotherapy types (p=0.276) and cycles (p=0.288) were not different between N-stage groups. Characteristics of the patients are presented in **Table 1**.

**Figure 1:**
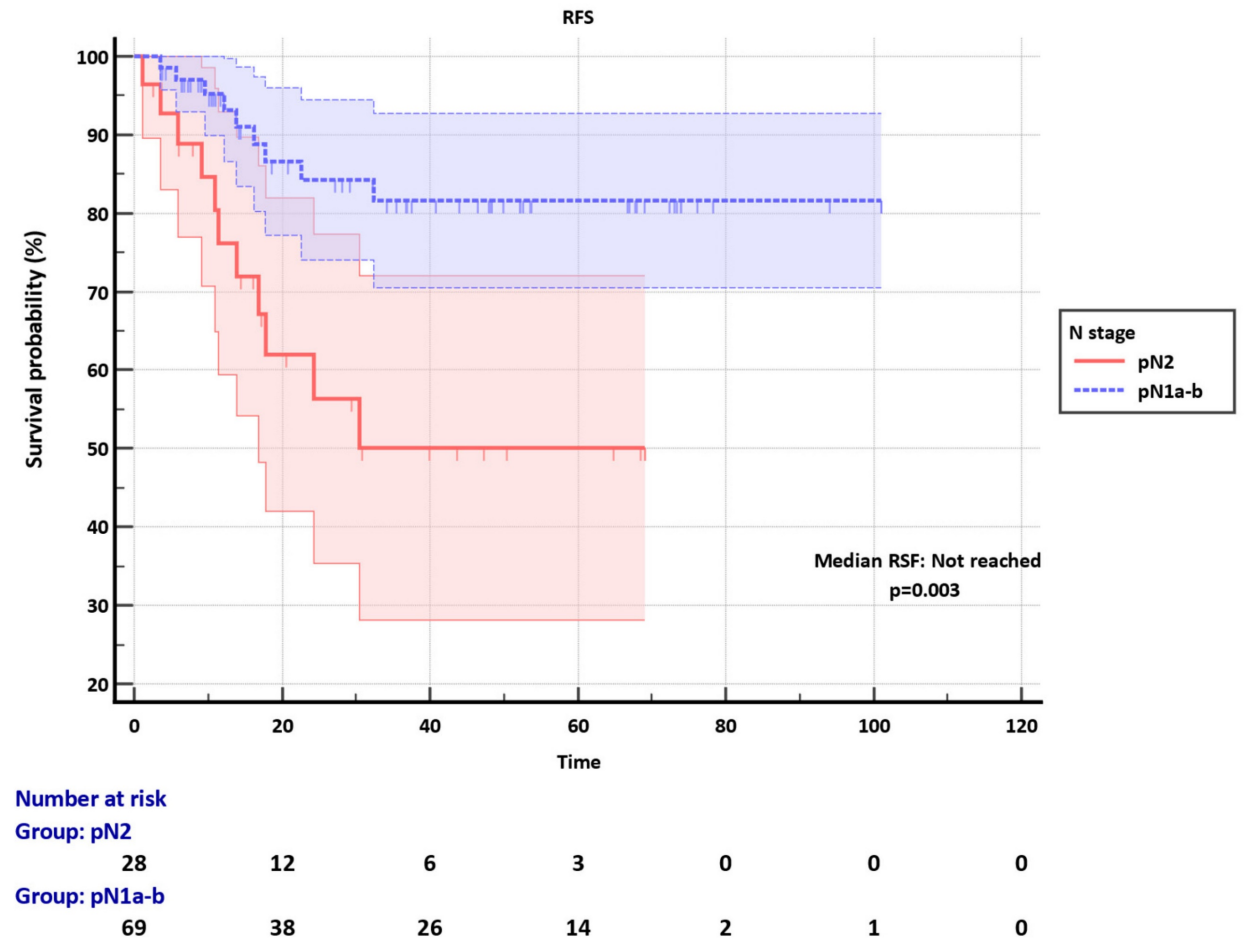
Recurrence-free survival (RFS) of pN1a-b and pN2 groups.

Median RFS was not reached in both pN1a-b and N2 groups during a median follow-up of 20.8 months (1.13-101.03). RFS was significantly better in the pN1a-b group compared to the pN2 (p=0.003) (**Figure 1**).

### Lymph node ratio (LNR) and prognosis

Median lymph nodes examined and involved in the entire study population were 22 (8-89) and 2 (1-17), respectively. Median lymph nodes examined were not different between pN1a-b and pN2 groups [21 (8-89) vs. 23.5 (10-57), p=0.151]. Median LNR was significantly higher in the pN2 group [0.07 (0.01-0.38) vs. 0.21 (0.04-0.84), p<0.001] (**Table 1**).

**Figure 2:**
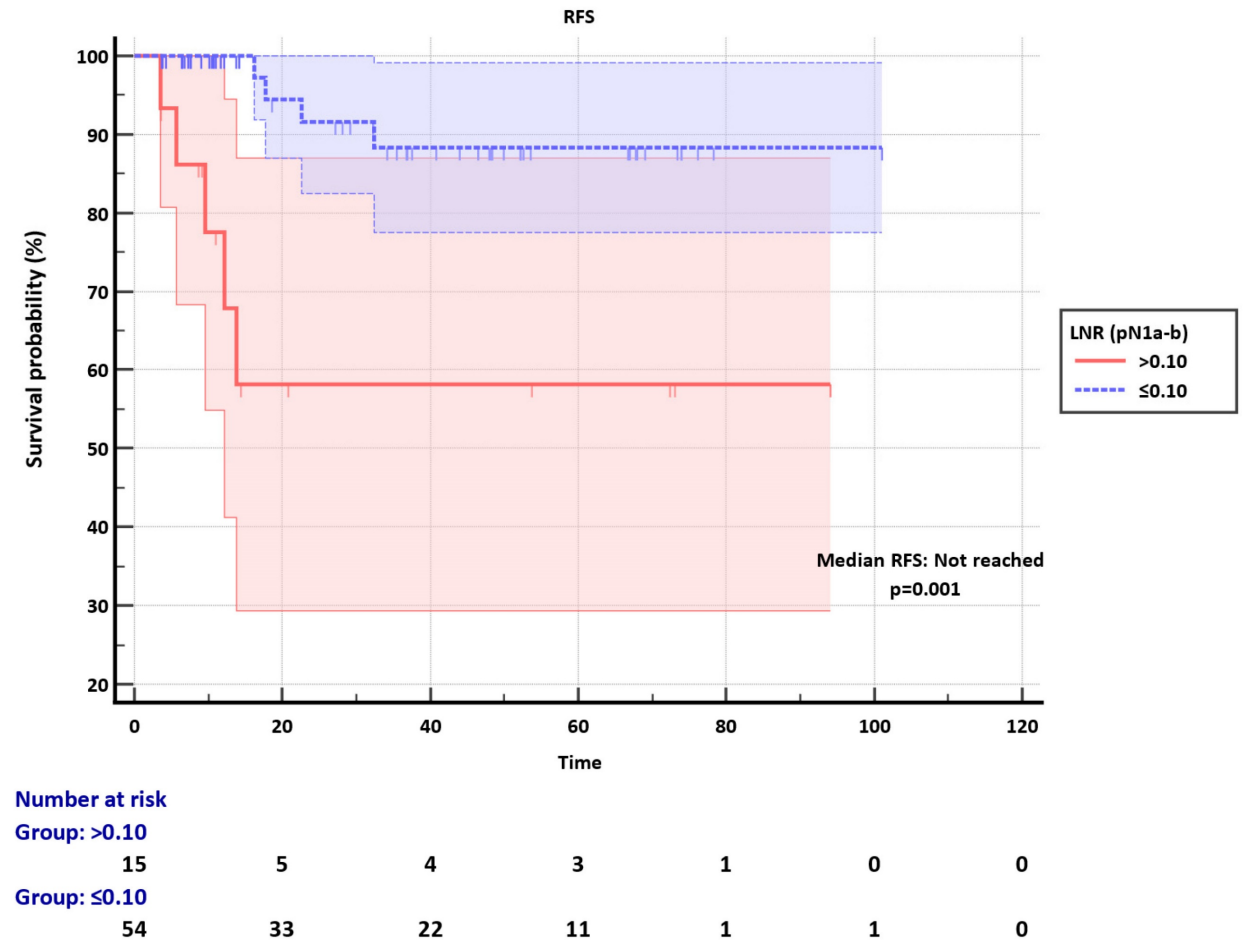
Recurrence-free survival (RFS) of pN1a-b group according to the LNR subgroups.

Among the pN1a-b group, the LNR cut-off was set as 0.10. Median RFS was not reached in both LNR ≤0.10 and LNR>0.10 subgroups, however, LNR significantly discriminated RFS in the pN1a-b (p=0.001) (**Figure 2**). The clinicopathological factors were not different between LNR ≤0.10 and LNR>0.10 subgroups (**Table S1).**

**Figure 3:**
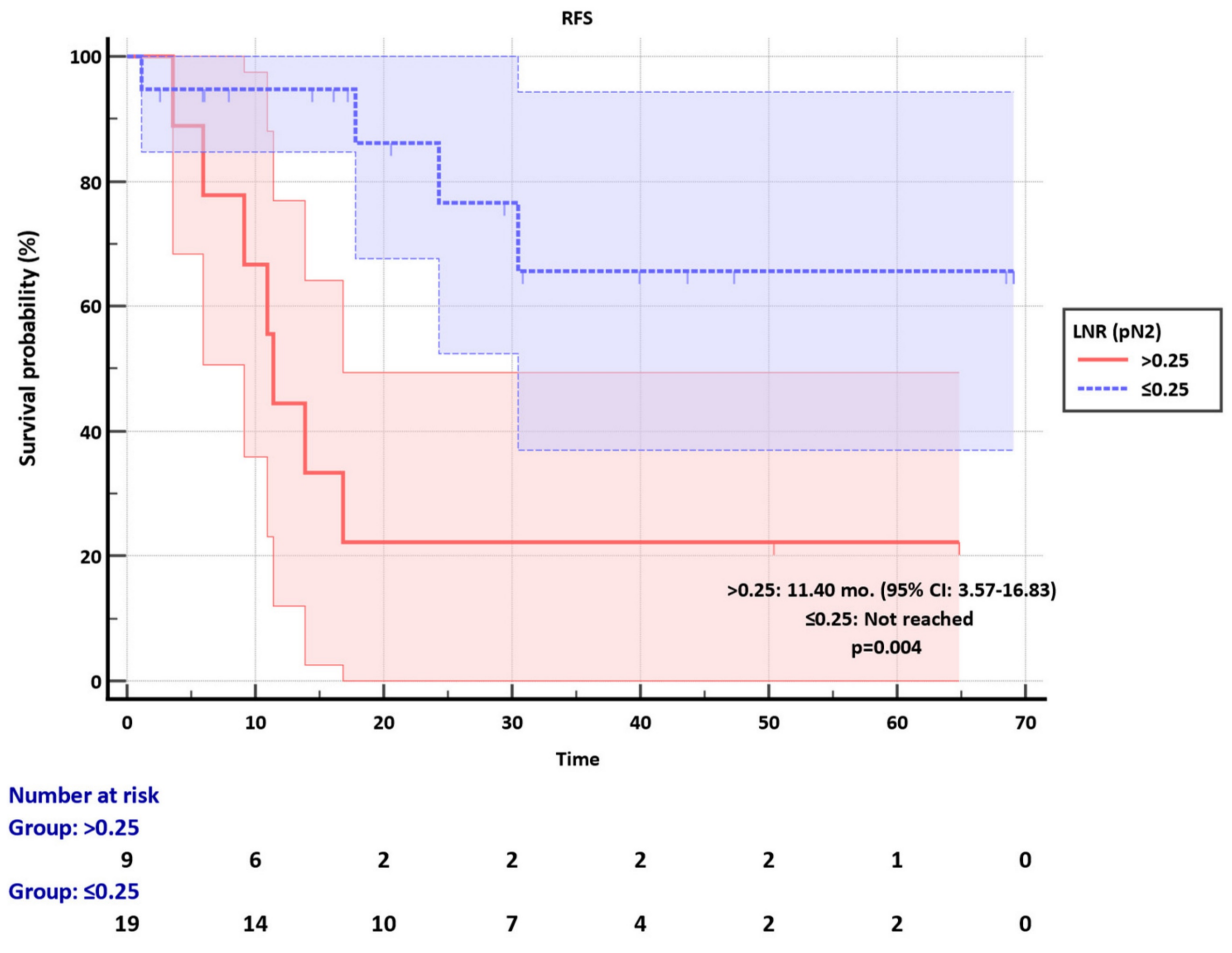
Recurrence-free survival (RFS) of pN2 group according to the LNR subgroups.

Among the pN2 group, the LNR cut-off was set as 0.25. Median RFS was not reached in the LNR ≤0.25 subgroup, whereas it was 11.40 months (95% CI: 3.57-16.83) in the LNR>0.25, significantly discriminating RFS (p=0.004) (**Figure 3**). The clinicopathological factors were not different between LNR ≤0.25 and LNR>0.25 groups (**Table S1**).

**Figure 4:**
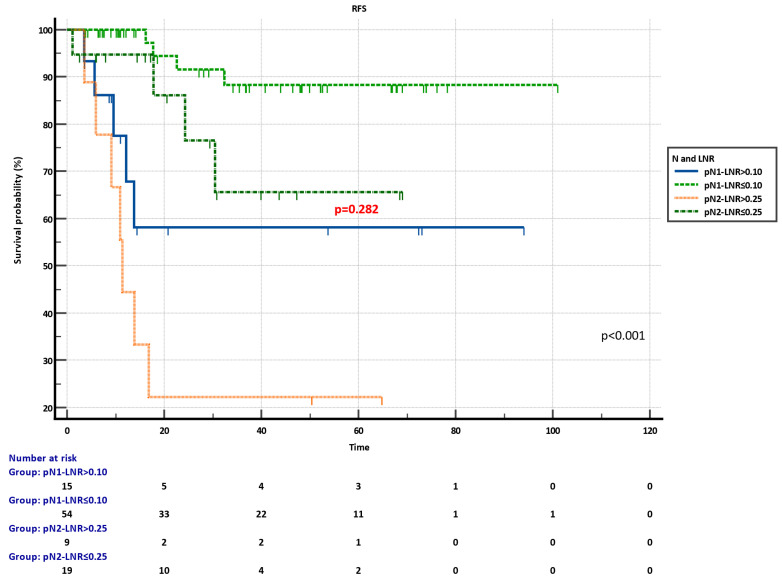
Recurrence-free survival (RFS) of combined pN-LNR groups.

Kaplan-Meier plots of combined pN-LNR groups revealed significant discrimination in RFS (p<0.001) (**Figure 4**). Moreover, the pN2-LNR≤0.25 group showed a tendency of better survival compared to the pN1-LNR>0.10 group, and RFS was not statistically different in these two subgroups (p=0.282) (**Figure 4**).

Variables that were significant in univariable analysis (mucinous component, RAS mutant status, N stage, and LNR) (**Table S2**) and variables different between pN1a-b and N2 groups (LVI, PNI, budding, MSI status) were included in multivariable Cox-regression analysis. Only LNR was significantly associated with RFS (p=0.023), whereas the pN stage did not remain significant (p=0.637) (**Table 2**).

## Discussion

This study showed that LNR is a prognostic factor and may add further prognostication in pN1a-b and pN2 stage III colon cancer. LNR revealed patient subgroups in pN1a-b and pN2 stages who may have similar prognoses despite different TNM N stages. Thus, our study suggests that after determining the TNM N stage of the disease in daily practice, considering LNR in individual patients may provide further opinions about the expected prognosis.

Patients with stage III colon cancer constitute a group in the localized disease where the adjuvant treatment strategy is critical. Although T and N stages are classified in detail, patients with stage III colon cancer show a heterogeneous course in terms of recurrence-free survival. Six months of adjuvant oxaliplatin-based chemotherapy have been standard for patients with stage III colon cancer; however, it is currently a debate since recent randomized trials have shown that 3-month chemotherapy may be non-inferior^13^. Although the patients with T4 or N2 tumors among stage III disease were considered high risk and recommended 6-month treatment^14^, further prognostic markers are needed to decide treatment duration in those patients. LNR, as shown in this study, may be considered an adjunct marker in treatment decisions.

In the present study, the optimal LNR cutoff values were determined to be 0.1 for pN1a-b and 0.25 for pN2. In a retrospective study conducted by Wang et al. on stage-III colon cancer patients operated between 2003 and 2008, the optimal cutoff values for the LNR were determined to be 1/14, 0.25, and 0.5, and patients were stratified into four groups. In the comparison made in terms of LNR, no survival difference was found in stage IIIA, while LNR and survival were found to be negatively correlated in the analysis made in the IIIB and IIIC subgroups^7^. A study of 19 French centers, comprising 250 patients, revealed an LNR cut-off of 0.1, and statistically significant differences were observed between the two groups in terms of three-year disease-free survival and overall survival^15^. In a study conducted in Denmark between 2003 and 2008 with 8,901 patients with stage III colon cancer, the LNR cut-off values (1/12, 1/4, and 1/2) were established as the LNR cut-off. The 5-year overall survival rates were 68.1%, 57.2%, 49.3%, and 32.4%, respectively. A statistically significant negative correlation was identified between LNR and survival (P < 0.0001)^16^. In a study of 26,181 patients, the LNR cutoff value was determined to be 0.4. The five-year cause-specific survival rates were 56% and 25%, respectively^17^. A further study divided patients into three LNR groups according to the cutoffs of 0.12 and 0.27 for LNR and observed that elevated LNR correlated with reduced OS and DFS. Furthermore, when the N1a group was stratified as low or high risk based on LNR, the 5-year OS was 78% in the low LNR group and 43% in the high LNR group^18^.

The results of our study indicate that there was no statistically significant difference between the recurrence-free survival (RFS) of pN1 patients with LNR>0.1 and pN2 patients with LNR≤0.25. This result indicates that the lymph node ratio, similar to the lymph node positivity, may be an important prognostic marker that warrants further consideration in prospective trials. The researchers of another multicenter study of 4172 patients proposed an alternative staging to the TNM using a combination of T stage and LNR, based on their results^12^. However, currently, there is no alternative staging that has global consensus. Thus, utilizing the LNR as an aid in daily practice is a more practical and pragmatic approach at the time being. Additionally, the removal of adequate and as much as possible lymph node sampling may be considered in surgery, to more precise the prognostication.

This study has several limitations. Firstly, the study has all the limitations of a retrospective design, such as limited control over sampling, nature and quality of the predictor variables, and missing data. Secondly, further studies with bigger sample sizes are needed. We could not differentiate further between pN1a and pN1b, and pN2a and pN2b, since the accuracy of analyses would substantially decrease. Finally, any certain recommendations in the management would require prospective randomized trials, however, LNR may still aid adjunctly.

## Conclusion

In conclusion, LNR discriminates prognostication in pN1a-b and pN2 stage-III colon cancer and reveals patient subgroups in pN1a-b and pN2 stages, who may have similar prognoses despite different TNM N stages. Until randomized controlled trials show proven recommendations, LNR may be considered and utilized in daily practice after determining pN1a-b and pN2 TNM stages in individual patients to assess prognosis further and decide treatment duration when additional marker is needed.

## Supplementary Material

Supplementary tables.

## Figures and Tables

**Table 1:** Characteristics of the study population and N-stage subgroups

	Entire study population (n=97)	pN1a-b (n=69)	pN2 (n=28)	P*
Age, median (min-max) (continuous variable)	67 (21-94)	67 (28-94)	66.5 (21-80)	0.574
Gender, n (%)				0.804
Male	57 (58.8)	40 (58)	17 (60.7)	
Female	40 (41.2)	29 (42)	11 (39.3)	
Diabetes, n (%)	19 (19.6)	13 (18.8)	6 (21.4)	0.771
Hypertension, n (%)	17 (17.5)	10 (14.5)	7 (25)	0.245
CAD, n (%)	12 (12.4)	8 (11.6)	4 (14.3)	0.740
Primary tumor location, n (%)				0.536
Right	40 (41.2)	26 (37.7)	14 (50)	
Transverse	4 (4.1)	3 (4.3)	1 (3.6)	
Left	53 (54.6)	40 (58)	13 (46.4)	
Urgent surgery, n (%)				0.781
No	87 (89.7)	62 (89.9)	25 (89.3)	
Obstruction	9 (9.3)	6 (8.7)	3 (10.7)	
Perforation	1 (1)	1 (1.4)	0 (0)	
pT stage, n (%)				0.834
2	3 (3.1)	2 (2.9)	1 (3.6)	
3	70 (72.2)	51 (73.9)	19 (67.9)	
4	24 (24.7)	16 (23.2)	8 (28.5)	
Tumor grade, n (%)				0.292
1	6 (6.2)	3 (4.3)	3 (10.7)	
2	68 (70.1)	53 (76.8)	15 (53.5)	
3	14 (14.4)	9 (13)	5 (17.9)	
UK	9 (9.3)	4 (5.9)	5 (17.9)	
Lymph nodes examined, median (min-max) (continuous variable)	22 (8-89)	21 (8-89)	23.5 (10-57)	0.151
Lymph nodes involved, median (min-max) (continuous variable)	2 (1-17)	1 (1-3)	5 (4-17)	<0.001
LNR, median (min-max) (continuous variable)	0.09 (0.01-0.84)	0.07 (0.01-0.38)	0.21 (0.04-0.84)	<0.001
Mucinous component, n (%)	20 (20.6)	10 (14.5)	10 (35.7)	0.019
LVI, n (%)	49 (50.5)	30 (43.5)	19 (67.9)	0.030
PNI, n (%)	33 (34)	18 (26.1)	15 (53.6)	0.010
Budding, n (%)	23 (23.7)	11 (15.9)	12 (42.9)	0.005
MSI-H, n (%)	5 (5.2)	1 (1.4)	4 (14.3)	0.025
RAS, n (%)				0.691
WT	28 (28.9)	19 (27.6)	9 (32.1)	
Mutant	9 (9.3)	5 (7.2)	4 (14.3)	
UK	60 (61.9)	45 (65.2)	15 (53.6)	
RAF, n (%)				0.351
WT	36 (37.1)	24 (34.8)	12 (42.9)	
Mutant	1 (1)	0 (0)	1 (3.6)	
UK	60 (61.9)	45 (65.2)	15 (53.5)	
ABO group, n (%)				0.689
AB	6 (6.2)	5 (7.2)	1 (3.6)	
A	49 (50.5)	36 (52.2)	13 (46.4)	
B	14 (14.4)	11 (15.9)	3 (10.7)	
O	19 (19.6)	12 (17.4)	7 (25)	
UK	9 (9.3)	5 (7.3)	4 (14.3)	
Rh, n (%)				0.206
Positive	80 (82.5)	60 (87)	20 (71.4)	
Negative	8 (8.2)	4 (5.8)	4 (14.3)	
UK	9 (9.3)	5 (7.2)	4 (14.3)	
Adjuvant chemotherapy, n (%)				0.276
FOLFOX	43 (44.3)	30 (43.5)	13 (46.4)	
XELOX	38 (39.2)	25 (36.2)	13 (46.4)	
Capecitabine	16 (16.4)	14 (20.3)	2 (7.2)	
Adjuvant chemotherapy cycle, median (min-max) (continuous variable)	8 (4-12)	10 (4-12)	8 (4-12)	0.288

Abbreviations: CAD: coronary artery disease, LNR: lymph node ratio, LVI: lymphovascular invasion, PNI: perineural invasion, WT: wild type, FOLFOX: Fluorouracil, leucovorin, and oxaliplatin, MSI-H: microsatellite instability-high, UK: unknown, XELOX: Capecitabine and oxaliplatin.

**Table 2:** Multivariable Cox regression analysis for RFS

Variable	HR (95% CI)	*P*
LNR (continuous variable)	90.59 (2.51-326.57)	0.023
pN stage (N2 vs N1a-b)	0.50 (0.03-8.43)	0.637
Mucinous component (Present vs absent)	0.41 (0.02-6.18)	0.524
RAS (Mutant vs WT)	0.14 (0.01-1.17)	0.070
LVI (Present vs absent)	1.27 (0.10-14.79)	0.848
PNI (Present vs absent)	5.07 (0.40-63.47)	0.208
Budding (Present vs absent)	0.58 (0.04-8.62)	0.695
MSI status (High vs low)	NC	0.992

Abbreviations: LNR: lymph node ratio, LVI: lymphovascular invasion, PNI: perineural invasion, WT: wild type, MSI-H: microsatellite instability-high
