# Supplementary material for: Lymph Node Ratio (LNR) Discriminates Prognostication in pN1a-b and pN2 Stage-III Colon Cancer
Source: J Cancer. 2025 Jan 1;16(4):1032–9. doi: 10.7150/jca.104336 (PMC11786040; doi:10.7150/jca.104336)
Supplement: Supplementary file 1 — Supplementary tables. [file jcav16p1032s1.pdf]

Supplementary

Tables

Table S1. Comparison of clinicopathological characteristics between LNR subgroups among pN1a-b and pN2 stages

Variable	pN1a-b<br>*p* for LNR ≤0.10 vs >10	pN2<br>*p* for LNR ≤0.25 vs >0.25
**Age**	0.351	0.482
**Gender**	0.857	0.657
**Diabetes**	0.897	0.944
**Hypertension**	0.679	0.815
**CAD**	0.812	0.409
**Primary tumor location**	0.155	0.672
**Urgent surgery**	0.821	0.963
**pT stage**	0.112	0.748
**Tumor grade**	**0.033**	0.848
**Mucinous component**	0.885	0.210
**LVI**	0.778	0.195
**PNI**	0.514	0.114
**Budding**	0.755	0.687
**MSI-H**	0.604	0.660
**RAS mutation**	0.126	0.522
**RAF mutation**	NC	0.657
**ABO groups**	0.642	0.915
**Rh**	0.876	0.251
**Adjuvant chemotherapy type**	0.618	0.208

Abbreviations: CAD: coronary artery disease, LVI: lymphovascular invasion, PNI: perineural invasion, MSI-H: microsatellite instability-high, NC: Not calculated

Table S2. Univariable Cox regression analysis for RFS

Variable	HR (95% CI)	P
**Age (Continous variable)**	0.97 (0.94-1.00)	0.127
**Gender, (Male vs. female)**	0.82 (0.34-2.00)	0.674
**Diabetes (Present vs absent)**	0.87 (0.25-2.98)	0.829
**Hypertension, (Present vs absent)**	1.88 (0.68-5.19)	0.221
**CAD, n (%)**	0.94 (0.21-4.08)	0.939
**Tumor location (Left vs right)**	0.79 (0.50-1.23)	0.300
**Urgent surgery (Obstruction vs. no)**	1.82 (0.77-4.32)	0.169
**T stage (T4 vs T3)**	1.77 (0.73-4.29)	0.203
**Lymph node examined (Continous variable)**	0.99 (0.96-1.03)	0.927
**Lymph node involved (Continous variable)**	1.21 (1.11-1.33)	**0.000**
**LNR (Continous variable)**	111.16 (17.35-711.89)	**0.000**
**N stage (pN2 vs pN1a-n)**	3.43 (1.42-8.32)	**0.006**
**Tumor grade (3 vs 2)**	0.71 (0.08-5.73)	0.535
**Mucinous component (Present vs absent)**	3.11 (1.29-7.51)	**0.012**
**LVI (Present vs absent)**	1.84 (0.73-4.62)	0.192
**PNI (Present vs absent)**	1.41 (0.58-3.42)	0.439
**Budding (Present vs absent)**	1.07 (0.41-2.79)	0.888
**MSI-H (Yes vs No)**	2.60 (0.75-9.03)	0.130
**RAS (Mutant vs WT)**	4.84 (1.35-17.25)	**0.015**
**ABO group**		
**A vs AB**	0.63 (0.14-2.90)	0.575
**B vs AB**	0.19 (0.01-2.13)	0.181
**O vs AB**	0.28 (0.04-2.05)	0.215
**Rh (Negative vs positive)**	0.51 (0.06-3.84)	0.515
**Perioperative treatment (Yes vs No)**	0.04 (0.00-55.20)	0.38
**Adjuvant chemotherapy (XELOX vs FOLFOX)**	1.42 (0.75-2.68)	0.276
**Adjuvant chemotherapy cycle (Continous variable)**	0.92 (0.78-1.08)	0.337

Abbreviations: CAD: coronary artery disease, LNR: lymph node ratio, LVI: lymphovascular invasion, PNI: perineural invasion, WT: wild type, FOLFOX: Fluorouracil, leucovorin, and oxaliplatin, MSI-H: microsatellite instability-high, UK: unknown, XELOX: Capecitabine and oxaliplatin.
